# Path Analysis of Factors Influencing Job Satisfaction Among Clinical Nurse Managers: A Cross‐Sectional Study

**DOI:** 10.1002/hsr2.73390

**Published:** 2026-10-13

**Authors:** Zhang WenXin, Liu Liping, Li Yue, Sun YuHong

**Affiliations:** ^1^ Nursing Department China–Japan Friendship Hospital China

**Keywords:** clinical nurse manager, job satisfaction, job stress, organizational support

## Abstract

**Background and Aims:**

Clinical nurse managers play a critical bridging role between hospital administration and frontline nursing staff, yet their job satisfaction remains understudied, particularly in Chinese healthcare contexts. Existing research often examines work stress and organizational support in isolation, overlooking their interrelationships. This study aimed to investigate the current status of job stress, job satisfaction, and perceived organizational support (POS) among clinical nurse managers, and to examine the mediating role of POS in the relationship between job stress and job satisfaction using path analysis.

**Methods:**

A cross‐sectional descriptive design was employed. A convenience sample of 192 clinical nurse managers from a tertiary Grade A hospital in Beijing completed online surveys during June–July 2024. Measures included the Minnesota Satisfaction Questionnaire (MSQ), the Nurse Work Stress Source Scale, and an Organizational Support Scale. Path analysis with bootstrapping was conducted using SPSS 27.0 and AMOS 21.0 to test direct, indirect, and total effects.

**Results:**

Overall job satisfaction was moderate to high (81.42 ± 10.34). Job satisfaction was positively correlated with POS and negatively correlated with work stress. The final structural model demonstrated good fit, explaining 62% of the variance in job satisfaction. Work stress directly negatively predicted job satisfaction (*β* = −0.315). POS directly positively predicted job satisfaction (*β* = 0.545). Importantly, POS significantly mediated the stress–satisfaction relationship, indicating that higher organizational support reduces the negative impact of work stress on satisfaction.

**Conclusion:**

Perceived organizational support directly enhances job satisfaction and partially buffers the detrimental effect of work stress among clinical nurse managers. Hospital administrators should prioritize resource allocation, developmental opportunities, and structural support—particularly for nursing team leaders and older managers—to improve job satisfaction and retention. Future research should employ longitudinal and multi‑center designs to establish causality and test context‑specific interventions.

## 1 Introduction

Clinical nurse managers occupy a pivotal position as the critical interface between administrative leadership and frontline nursing staff, shouldering direct accountability for the oversight, coordination, and seamless execution of daily clinical operations. Their comprehensive role spans vital organizational functions, including strategic coordination, effective communication, and transformative leadership within complex healthcare environments [1]. In the modern healthcare landscape, marked by escalating complexity and evolving challenges, clinical nurse managers have become principal architects of organizational success in healthcare settings. These professionals serve as vital catalysts in cultivating supportive work environments that not only enable the consistent delivery of safe, high‐quality patient care but also drive the achievement of strategic organizational goals. Their dual focus on workforce well‐being and institutional objectives positions them as indispensable agents of positive change within healthcare systems. The responsibilities of clinical nurse managers extend across critical domains, including patient care management, quality improvement initiatives, professional development programs, talent cultivation, and workforce retention strategies [2]. Operating within severe resource constraints, clinical nurse managers face the persistent challenge of maintaining exceptional care standards, which inevitably results in considerable occupational stress.

Organizational support theory posits that employees' perception of institutional backing significantly influences their work engagement and stress levels [3, 4]. Empirical evidence suggests that inadequate organizational support adversely affects nurse managers' job satisfaction [5]. Recent studies have identified a strong positive correlation between clinical nurse managers' perceived team support and job satisfaction, while demonstrating an inverse relationship between stress levels and job satisfaction [6]. The critical importance of job satisfaction among clinical nurse managers for effective care management has been well established, with work‐related stress identified as a significant determinant of satisfaction levels [7, 8].

While substantial research has been conducted on nurses' job satisfaction and its determinants [9], there remains a paucity of studies specifically examining clinical nurse managers' satisfaction and its influencing factors. Existing research has often investigated these factors in isolation, leaving their interrelationships and combined impact on job satisfaction inadequately understood. This study seeks to address these gaps by investigating the current status of job stress, job satisfaction, and perceived organizational support (POS) among clinical nurse managers. Furthermore, this study aims to systematically examine the mediating role of organizational support in the relationship between job stress and job satisfaction by employing advanced path analysis methods. By uncovering these intricate interrelationships, the research offers valuable insights that deepen our understanding of these critical workplace dynamics. Ultimately, the findings are expected to inform the development of more effective strategies to enhance nurse managers' well‐being and optimize organizational outcomes, thereby fostering a more supportive and productive healthcare work environment.

## 2 Research Gaps and Study Justification

While extant literature has established associations between nurse satisfaction and individual/demographic factors [9], critical limitations persist:

- *Conceptual Fragmentation*: Previous studies have examined stressors and supports in isolation, neglecting the synergistic effects of multiple variables.
- *Methodological Constraints*: Limited use of advanced statistical techniques (e.g., path analysis) to capture complex mediating mechanisms.
- *Cultural Specificity*: Insufficient research on Chinese healthcare contexts, where hierarchical management structures and resource allocation paradigms differ markedly from Western systems.

This study addresses these gaps by:

- Employing structural equation modeling (SEM) to examine the direct and indirect effects of POS in the stress–satisfaction relationship.
- Incorporating multi‐dimensional measures of organizational support (instrumental, emotional, informational).
- Focusing on a Chinese sample to advance cross‐cultural comparative understanding.

## 3 Methods

### 3.1 Study Design

This research employed a descriptive study design with a cross‐sectional approach. The primary objective was to explore associations and correlations among variables.

### 3.2 Sample and Data Collection

Eligible participants included nursing leaders with more than 1 month of clinical management experience who provided consent. Exclusions applied to administrative managers, staff absent for more than 3 months, and those primarily engaged in research or teaching. During June–July 2024, clinical nurse managers from a tertiary Grade A hospital in Beijing were recruited through convenience sampling. After receiving study information and providing consent, participants completed a mandatory online survey containing demographic items and standardized scales; all fields were required for submission. This research was approved by the hospital's Institutional Review Board.

### 3.3 Survey Tool

Job satisfaction was assessed using the Minnesota Satisfaction Questionnaire short form (MSQ [10]), a 20‐item scale with three dimensions (intrinsic, extrinsic, and general satisfaction) rated on a 5‐point Likert scale (1 = very dissatisfied to 5 = very satisfied). With established reliability (Cronbach's *α* = 0.89–0.95 [11]), the scale demonstrated excellent internal consistency in this study (α=0.948).

Work‐related stress was assessed using the 35‐item Nurse Work Stress Source Scale [12], covering five dimensions: professional issues, workload, work environment, patient care, and interpersonal difficulties. Using a 4‐point Likert scale (1 = no stress to 4 = severe stress; total score range 35–140), the scale showed excellent reliability (α=0.98) in previous studies and good reliability in this study (α=0.86).

### 3.4 Data Analysis

Data analysis was performed using IBM SPSS 27.0 for descriptive statistics and correlation analysis [13], and AMOS 21.0 for SEM.

#### 3.4.1 Measurement Model

Before testing the structural model, a confirmatory factor analysis was conducted to assess the reliability and validity of the latent variables. The measurement model demonstrated adequate fit: *χ* ^2^/df = 1.92, CFI = 0.965, TLI = 0.958, RMSEA = 0.048. Composite reliability for all constructs exceeded 0.80, and average variance extracted (AVE) exceeded 0.50, confirming convergent validity. Discriminant validity was established as the square roots of AVEs were greater than the inter‐construct correlations.

#### 3.4.2 Structural Model and Mediation Testing

Path analysis was employed to test the hypothesized relationships (Figure 1). Model fit was assessed using multiple indices: chi‐square, CFI, GFI, and RMSEA. The model was refined based on modification indices (MIs) only when theoretically justified. Specifically, a path from “position” to “job satisfaction” was added, which substantially improved model fit without compromising theoretical parsimony. The significance of direct and indirect effects was tested using bias‐corrected bootstrapping with 5000 resamples to generate 95% confidence intervals (CIs). Indirect effects were considered significant if the 95% CI did not include zero. The variance explained (*R* ^2^) for endogenous variables was also calculated.

**Figure 1:**
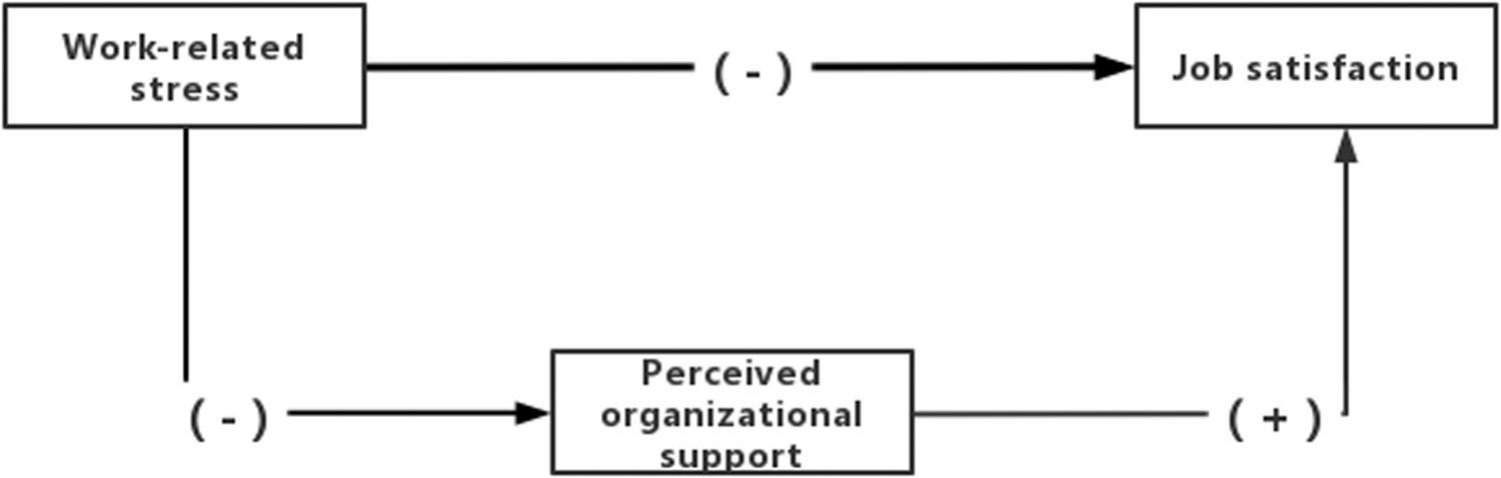
Hypothetical model of clinical nurse managers' satisfaction, organizational support, and stressors.

## 4 Results

All 192 clinical nurse managers completed the survey (100% response rate). Use means and standard deviations (SDs) for normally distributed data. Job satisfaction scores were 81.42 ± 10.34, work stress scores were 79.31 ± 20.14, and organizational support scores were 45.66 ± 6.99. Table 1 shows participant demographics and subgroup comparisons of job satisfaction.

**Table 1:** Comparison of job satisfaction in clinical nurse managers with different characteristics (*n* = 192).

Variables	*n* (%)	Score of job satisfaction	*t*/*F* Value	*p*
Age (years)			1.531	0.219
≤ 35	56 (29.17)	78.91 ± 9.31		
36–45	102 (53.13)	82.95 ± 10.34		
≥ 46	34 (17.70)	81.35 ± 10.27		
Gender			2.427	0.016
Male	6 (3.12)	71.33 ± 10.45		
Female	186 (96.88)	81.74 ± 10.34		
Professional titles			2.589	0.078
Senior nurse	27 (14.06)	77.52 ± 10.61		
Supervisor nurse	159 (82.81)	81.93 ± 10.34		
Associate chief nurse and above	6 (3.13)	85.33 ± 9.26		
Work experience (years)			1.356	0.098
≤ 10	30 (15.62)	77.23 ± 10.41		
11–20	107 (55.73)	82.83 ± 10.22		
≥ 21	55 (28.65)	80.95 ± 10.36		
Tenure of office(years)			0.04	0.947
≤ 5	126 (65.63)	80.95 ± 10.33		
6–10	44 (22.91)	81.64 ± 9.74		
≥ 11	22 (11.46)	83.64 ± 9.74		
Position			5.339	0.006
Charge nurse	99 (51.57)	79.40 ± 10.37		
Head nurse	78 (40.62)	82.82 ± 10.32		
Supervisor	15 (7.81)	87.40 ± 9.35		

### 4.1 Descriptive Statistics and Correlation Analysis of Study Variables

Descriptive statistics for the three primary measures—the Job Satisfaction Scale, Organizational Support Scale, and Nurse Stress Source Scale—are presented in Table 2, reflecting responses from the clinical nurse manager cohort. Subsequent bivariate correlation analysis revealed significant associations between job satisfaction and other study variables, with detailed statistical relationships documented in Table 3.

**Table 2:** Descriptive analysis of the study variables (*n* = 192).

Variables	Minimum values	Maximum values	Mean	Standard deviation	Skewness	Kurtosis
Job satisfaction	38.00	100.00	81.42	10.34	−0.47	1.50
Perceived organization support	23.00	63.00	45.66	6.99	−0.46	0.41
Stressors	35.00	130.00	79.31	20.14	−0.19	−0.23

**Table 3:** Correlation analysis of various research variables (*n* = 192).

Variables	Job satisfaction	Perceived organization support	Stressors	Age	Years of service	Tenure of office (years)
Job satisfaction	1					
Perceived organization support	0.712**	1				
Stressors	−0.415**	−0.291**	1			
Age	0.092*	0.052	−0.068	1		
Years of service	0.093*	0.044	−0.08	0.855**	1	
Tenure of office (years)	0.113*	0.019	−0.014	0.623**	0.668**	1
Position	0.255**	0.244**	−0.140**	0.333**	0.278**	0.364**

* *p* < 0.05;

** *p* < 0.001.

### 4.2 Pathway Analysis of Factors Affecting Job Satisfaction Among Clinical Nurse Managers

Based on organizational support theory and a review of relevant literature, this study identified position, technical title, and source of stress as external variables. Organizational support was considered a mediating variable, while job satisfaction was classified as an internal variable for hypothesis formulation. A validation analysis was conducted on the model, which was subsequently revised using an MI, followed by an evaluation of model fit. The final path analysis model is illustrated in Figure 2. The modified model fit indices for the factors influencing job satisfaction among clinical nurse managers were as follows: chi‐square value = 3.71, degrees of freedom = 2, *p* = 0.156 (> 0.05), CFI = 0.991 (> 0.90), GFI = 0.994 (> 0.90), and RMSEA = 0.047 (< 0.05). The final model also presented the direct, indirect, and total effects of the factors affecting job satisfaction among clinical nurse managers, as detailed in Table 4. Factor loadings are presented in Table 5.

**Figure 2:**
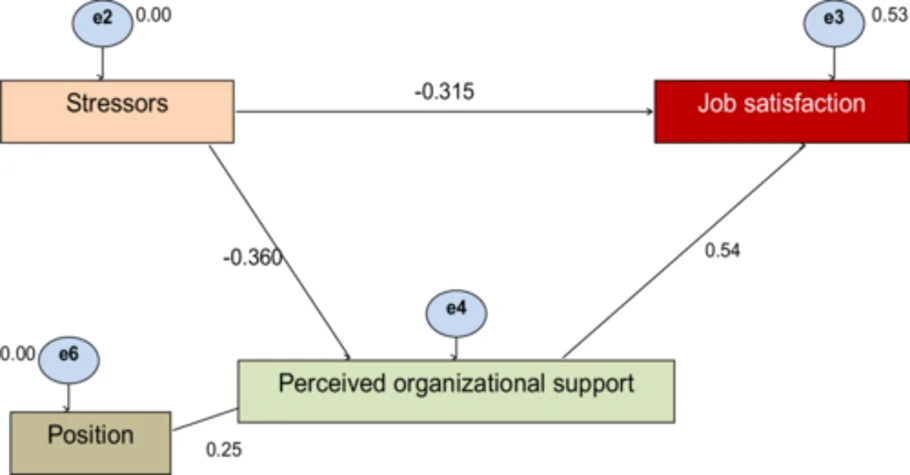
Path diagram of factors influencing job satisfaction among clinical nurse managers.

**Table 4:** Direct, indirect, and total effects of the modified model of factors influencing job satisfaction among clinical nurse managers.

Factors	Position			Stressors			Perceived organization support		
	D	ID	T	D	ID	T	D	ID	T
Job satisfaction	0.000	0.135	0.135	−0.315	−0.196	−0.511	0.545	0.000	0.545

Abbreviations: D, direct effect; ID, indirect effect; T, total effect.

**Table 5:** Path model results: Standardized coefficients.

Latent variable	Indicator	Standardized factor coefficients
Perceived organizational support	Stressors	−0.360
	Position	0.247
Job satisfaction	Stressors	−0.315
	Perceived organizational support	0.545

#### 4.2.1 Model Fit

The final structural model exhibited a good fit with the data: χ^2^ = 3.71, df = 2, *p* = 0.156; CFI = 0.991; GFI = 0.994; RMSEA = 0.047. The model accounted for a substantial proportion of variance in job satisfaction, with an *R* ^2^ value of 0.62 (Table 6), indicating that the model explains 62% of the variance in the primary outcome.

**Table 6:** Variance explained (*R* ^2^) in the modified path model.

Endogenous variable	*R* ^2^	Predictors	Direct effect (*β*)	Indirect effect (*β*)	95% CI (indirect)
Job satisfaction	0.62	Stressors	−0.315	−0.196	[−0.312, −0.098]
		POS	0.545	—	—
		Position	—	0.135	[0.045, 0.247]

#### 4.2.2 Mediation Effects

The standardized direct, indirect, and total effects are presented in Table 4. The bootstrap analysis revealed that POS significantly mediated the relationship between job stress and job satisfaction (indirect effect = −0.196, 95% CI [−0.312, −0.098]), supporting the hypothesized mediation model.

## 5 Discussion

The majority of clinical nurse managers in the study were female, accounting for 96.88% of the sample. The age distribution fell predominantly in the 36–45 year range. In addition, 82.81% of the participants were supervising nurses. Notably, 55.73% of managers had been in their positions for 11 years, while 28.65% had served for 21 years or more. Of those surveyed, 51.57% held the title of nursing team leader, 40.62% were head nurses, and 7.81% were department head nurses. Overall, clinical nurse managers reported high levels of job satisfaction; however, those aged 46 and older, male managers, and group leaders showed lower levels of satisfaction compared to their counterparts. Differences in satisfaction levels across age groups, genders, and positions were statistically significant. The reduced satisfaction among the older age group may be attributed to factors such as decreased energy, increased workload, and heightened work pressure, particularly within the context of the nursing vertical management model employed by the surveyed hospital, which operates under the “attending physician responsibility system” [14]. Under the management model of unified bed allocation throughout the hospital [15], numerous patients are admitted across various departments. This necessitates that nursing team leaders possess an optimized multidisciplinary nursing knowledge structure characterized by both breadth and sufficient depth. Following the elimination of traditional main shifts under the vertical care management model, nurses are reassigned to attending care teams as team leaders. One potential reason for the low satisfaction levels among nursing team leaders may be that these roles are filled by senior nurses who experience high levels of work pressure. In addition, the position is recognized by the department rather than appointed by the hospital, leading to responsibilities that may not align with formal recognition. Furthermore, the low satisfaction reported by male nursing managers could be attributed to relatively modest levels of work achievement and social recognition associated with the nursing role. The nursing department should prioritize the job satisfaction of male nurses, particularly those aged 46 and older, as well as nursing team leaders, and implement proactive measures to enhance work motivation [16].

Correlation analysis showed that job satisfaction among clinical nurse managers was positively correlated with organizational support and employment status, with correlation coefficients of 0.712 and 0.255, respectively. Conversely, job satisfaction was negatively correlated with work pressure, with a correlation coefficient of −0.415.

Based on organizational support theory, a hypothetical model was constructed. Following MI analysis, a path to position was added, resulting in an adapted model with fit indices meeting the desired criteria. Path analysis revealed that the source of work stress for nursing managers directly affected job satisfaction, with a path coefficient of −0.315, indicating that stress accounted for 31.5% of the variance in job satisfaction. In addition, sources of stress had a negative effect on POS, with a path coefficient of −0.360, and exerted an indirect effect of −0.196 on job satisfaction through POS. This suggests that lower work pressure is associated with higher job satisfaction, and that organizational support is associated with lower work pressure, which in turn is associated with higher satisfaction. Furthermore, organizational support directly influenced various aspects of job satisfaction, with a path coefficient of 0.545, indicating that organizational support explained 54.5% of the variance in managers' job satisfaction, consistent with the findings of similar research by Simpson et al. [17]. In addition, the manager's position indirectly affected job satisfaction through the provision of organizational support, suggesting that managers at different levels may experience varying levels of support. Therefore, it is essential to focus on the organizational support provided to nursing team leaders and head nurses to enhance their job satisfaction. Adequate organizational support, which includes sufficient opportunities for development, resources, and personnel, will contribute to improved job satisfaction among managers [18]. Similar studies have demonstrated that organizational support is a critical element of a healthy work environment and serves as a predictor of enhanced productivity, job satisfaction, and retention. This underscores the significance of POS for clinical nurse managers [19]. Due to the cross‐sectional design, the reported path coefficients should be interpreted as associations consistent with the proposed theoretical model, not as causal effects. Longitudinal or experimental designs are needed to establish causality.

The findings of this study can be further understood within the context of the Job Demands‐Resources (JD‐R) model [20]. Within this framework, job stress (e.g., workload, patient care complexities) can be conceptualized as a job demand that depletes energy and contributes to burnout, thereby negatively affecting job satisfaction. Conversely, POS (e.g., developmental opportunities, resource availability) functions as a job resource that buffers the impact of high demands and fosters engagement. The significant negative direct path from stress to satisfaction, coupled with the positive indirect path through organizational support, empirically demonstrates the JD‐R model's core proposition: resources can mitigate the detrimental effects of demands on employee outcomes. By integrating organizational support theory with the JD‐R model, our results suggest that hospitals seeking to enhance nurse manager retention should focus not only on reducing demands but, more importantly, on strengthening organizational support as a pivotal job resource.

## 6 Conclusion

Clinical nurse managers play a critical role in influencing patient outcomes, nursing staff retention, and various nursing specialties. In the Chinese healthcare context, where hierarchical authority and organizational endorsement carry substantial weight [21], perceived support from the hospital may be a more potent predictor of job satisfaction than in more individualistic or flatter organizational cultures. This cultural nuance represents an important extension of organizational support theory. However, the career development and job satisfaction of clinical nurse managers themselves should not be overlooked. This study demonstrates that organizational support has a direct impact on the job satisfaction of clinical nurse managers. Hospital administrators must prioritize resource allocation and structural reforms to mitigate burnout and retain critical leadership talent. Future research should focus on developing context‐specific interventions to optimize POS and improve clinical nurse managers' well‐being.

### 6.1 Limitations

This study has several limitations. First, convenience sampling from a single tertiary Grade A hospital in Beijing limits generalizability. Findings may not apply to secondary or community hospitals, hospitals in other regions of China, or healthcare systems outside China. Second, the sample size (*n* = 192), while adequate for the path analysis conducted, was insufficient for more complex subgroup analyses (e.g., by department or manager level). Third, the cross‑sectional design precludes causal inference; all path coefficients should be interpreted as associations consistent with the theoretical model, not causal effects. Fourth, all data were self‑reported at a single time point, raising the possibility of common method bias, although a post‑hoc Harman's single‑factor test indicated no dominant factor. Fifth, convenience sampling may have introduced selection bias, as highly stressed or disengaged managers might have opted out. Finally, while we used validated scales, the Nurse Work Stress Source Scale was developed in 2000 and may not fully capture contemporary stressors (e.g., electronic health record burden). Future multi‑center, longitudinal studies using mixed methods are needed to confirm and extend our findings.

## Author Contributions

The study was conceptualized and designed by Zhang WenXin and Sun YuHong. Li Yue was responsible for data collection. Liu Liping conducted the data analysis. Zhang WenXin drafted the initial manuscript. Sun YuHong contributed to the critical revision and editing of the manuscript. All authors (Zhang WenXin, Liu Liping, Li Yue, Sun YuHong) participated in reviewing and approving the final manuscript. All authors take responsibility for the accuracy and integrity of all aspects of the work.

All authors have read and approved the final version of the manuscript. Zhang WenXin had full access to all of the data in this study and takes complete responsibility for the integrity of the data and the accuracy of the data analysis.

## Funding

The authors have nothing to report.

## Ethics Statement

This study was conducted as a descriptive, cross‐sectional survey. The Institutional Review Board of China–Japan Friendship Hospital approved this study (Approval No. 2023‐KY‐267), and all participants provided informed consent prior to data collection. Participation was voluntary, and informed consent was electronically obtained from all participants after they were provided with detailed study information. Data were collected anonymously via a secure web‐based survey platform in June–July 2024. The study employed convenience sampling of clinical nurse managers from a single tertiary hospital in Beijing, which may limit the generalizability of the findings. All measurement tools used were standardized and validated scales. Data analysis was performed using IBM SPSS 27.0 and AMOS 21.0. There are no conflicts of interest to declare. The dataset generated and analyzed during this study is not publicly available to protect participant confidentiality, but may be available from the corresponding author upon reasonable request.

## Conflicts of Interest

The authors declare no conflicts of interest.

## Transparency Statement

The lead/corresponding author, Zhang WenXin, affirms that this manuscript is an honest, accurate, and transparent account of the study being reported; that no important aspects of the study have been omitted; and that any discrepancies from the study as planned have been explained.

## Data Availability

The data that support the findings of this study are available from the corresponding author upon reasonable request, subject to institutional ethical approval and participant confidentiality agreements.
